# Validation of the Hidradenitis Suppurativa Investigator Global Assessment: A Novel Hidradenitis Suppurativa–Specific Investigator Global Assessment for Use in Interventional Trials

**DOI:** 10.1001/jamadermatol.2023.0797

**Published:** 2023-04-26

**Authors:** Amit Garg, Carla Zema, Valerie Ciaravino, Robert Rolleri, Luke Peterson, Llenalia Garcia, Tyler Massaro, Gregor B. E. Jemec, Joslyn S. Kirby, Linnea Thorlacius, John R. Ingram

**Affiliations:** 1 Donald and Barbara Zucker School of Medicine at Hofstra/Northwell, New Hyde Park, New York; 2 Zema Consulting, Madison, Alabama; 3 UCB Pharma, Colombes, France; 4 UCB Pharma, Raleigh, North Carolina; 5 UCB Pharma, Slough, United Kingdom; 6 Department of Dermatology, Zealand University Hospital, Roskilde, Denmark; 7 Department of Dermatology, Penn State Milton S. Hershey Medical Center, Hershey, Pennsylvania; 8 Division of Infection and Immunity, Cardiff University, University Hospital of Wales, Heath Park, Cardiff, United Kingdom

## Abstract

**Importance:**

Few simplified instruments exist for use in hidradenitis suppurativa (HS) trials.

**Objective:**

To assess psychometric properties of the Hidradenitis Suppurativa Investigator Global Assessment (HS-IGA) score using a clinical trial data set.

**Design, Setting, and Participants:**

This retrospective analysis of a phase 2 randomized double-blind, placebo-controlled, active-reference arm trial (UCB HS0001) included adults with moderate-to-severe HS.

**Exposures:**

Trial participants were randomized at baseline to receive bimekizumab, adalimumab, or placebo.

**Main Outcomes and Measures:**

The HS-IGA score at prespecified time points up to 12 weeks after randomization.

**Results:**

The HS-IGA score showed strong convergent validity with IHS4 and HS-PhGA scores at baseline (Spearman correlation, 0.86 [*P* < .001] and 0.74 [*P* < .001], respectively) and at week 12 (Spearman correlation, 0.73 [*P* < .001] and 0.64 [*P* < .001], respectively). The HS-IGA scores assessed during predosing visits at screening and baseline showed good test-retest reliability (intraclass correlation coefficient [ICC] = 0.92). At week 12, HS-IGA responders were significantly associated with HiSCR-(50/75/90) responders (χ^2^ = 18.45; *P* < .001; χ^2^ = 18.11; *P* < .001; and χ^2^ = 20.83; *P* < .001, respectively). The HS-IGA score was predictive of HiSCR-50/75/90 and HS-PhGA response at week 12 (AUC, 0.69, 0.73, 0.85, and 0.71, respectively). However, the HS-IGA as a measure of disease activity showed low predictive validity with patient-reported outcomes at week 12.

**Conclusions and Relevance:**

The HS-IGA score demonstrated good psychometric properties compared with existing measures and may be considered for use as an end point in clinical trials for HS.

## Key Points

**Question:**

Is the Hidradenitis Suppurativa Investigator Global Assessment (HS-IGA) a valid end-point measure for clinical trials in patients with hidradenitis suppurativa (HS?)

**Findings:**

In this secondary analysis of the UCB phase 2 randomized clinical trial that included 88 adults who received study treatment, The Hidradenitis Suppurativa Investigator Global Assessment (HS-IGA) demonstrated good convergent validity with IHS4 and HS-PhGA, strong test-retest reliability, and responsiveness to change when compared to HiSCR. The HS-IGA score was associated with accurate Hidradenitis Suppurativa Clinical Response (with HiSCR scores indicating 50%, 75%, and 90% reduction from baseline) and HS-PhGA clear or minimal response at week 12.

**Meaning:**

The HS-IGA demonstrated good psychometric properties compared with existing measures and may be considered for use as an end point in clinical trials for patients with HS.

## Teaser

This secondary analysis of data from a phase 2 randomized clinical trial assesses the psychometric properties of the Hidradenitis Suppurativa Investigator Global Assessment score.

## Introduction

Hidradenitis suppurativa (HS) is a potentially debilitating disease that disproportionately affects women and those who are Black.^1,2^ Given the symptoms of pain and drainage related to the disease, HS is known to have substantial effects on general health-related and skin-specific quality of life (QoL).^3^ Disease morbidity for patients is likely exacerbated by a 10-year mean delay between onset of symptoms and diagnosis.^3^ Hidradenitis suppurativa has also been linked to considerable comorbidity burden^3,4^ and increased mortality risk.^5,6^

It has been shown^3^ that half of patients with HS are dissatisfied with current treatments due to perceived poor efficacy. Not surprisingly, nearly half of patients with HS express low optimism for satisfactory control of symptoms.^3^ Although there is growing interest in drug development, treatment represents the greatest unmet need in HS. Relative absence of simple severity and response measures for use in trials may hinder drug development.^7^ There is also a need for feasible instruments applicable in clinical settings.

To address this fundamental gap, there is a parallel initiative to develop a core measures set for HS trials with the goal of standardizing valid and reliable measurement of disease severity and treatment response and of comparing effectiveness. The Hidradenitis Suppurativa Core Outcomes Set International Collaboration (HiSTORIC) has established the core domain set (*what to measure*) in HS^8^ and has highlighted considerable challenges related to the question of *how to measure* disease activity reliably.^9^ The global assessment represents 1 of 6 HiSTORIC core domains. The Hidradenitis Suppurativa Investigator Global Assessment (HS-IGA) has been proposed as a measure to fulfill the global assessment domain, and to overcome limitations in existing measures used in clinical trials.

Recently, HiSTORIC described the development and initial validation of the HS-IGA in measuring disease activity and responsiveness to interventions.^10^ The construct was developed using data from 2 replicate phase 3 randomized clinical trials (PIONEER I [NCT01468207] and PIONEER II [NCT01468233])^11^ and an iterative process involving input from experts, patient research partners, and methodologists in HiSTORIC over 2 years. The purpose of the current study was to assess psychometric properties of HS-IGA using a separate, and more recent, trial data set.

## Methods

Data from the UCB HS0001 phase 2 study evaluating treatment (NCT03248531) were used to assess psychometric properties of the HS-IGA.^12^ This study involved secondary analysis of deidentified clinical trial data and did not require institutional review board review or written informed consent.

### Measures and Outcomes

The HS-IGA is scored as a number between 0 and 5 based on the sum of abscess, nodule (inflammatory and non-inflammatory), and fistula (draining and nondraining) counts, in either the upper- or lower-body regions, whichever is greater at the time of assessment. As such, the region used for scoring at baseline may not be the same region used at a given postbaseline visit. Papules, plaques, pustules, comedones, and scars are not counted in the score (Table 1). Response was defined as a 2-point improvement (reduction) in score relative to baseline, which is consistent with US Food and Drug Administration response definitions for Physician Global Assessment and Investigator Global Assessment (PGA/IGA) measures in dermatologic conditions. Responder status was calculated for all postbaseline visits.

**Table 1.** Hidradenitis Suppurativa Investigator Global Assessment (HS-IGA) Score^a^

HS-IGA Score	Lesion count
0	0-1
1	2-5
2	6-10
3	11-15
4	16-20
5	>20

^a^ The HS-IGA score is scored as a number between 0 and 5 based on the sum of abscess (A), fistula (F) (draining and nondraining), and nodule (N) (inflammatory and noninflammatory) count, either in the upper-body region or lower-body region, whichever is greater at the time of assessment. The region used for scoring at baseline may not be the same region used at a given postbaseline visit. Response is defined as at least a 2-point reduction in HS-IGA score relative to baseline. The HS-IGA score is Copyrighted by Amit Garg, John Ingram, Linnea Thorlacius, Gregor Jemec and is used here with permission.

Clinician-reported outcome (ClinRO) measures of HS severity considered for comparison included the Hidradenitis Suppurativa Clinical Response (HiSCR),^13^ International Hidradenitis Suppurativa Severity Score System (IHS4),^14^ and the HS Physician Global Assessment (HS-PhGA).^15^ The HiSCR, which is the current primary end point in interventional trials for HS, is defined as at least a 50% reduction in the total abscess and inflammatory nodule count from baseline, with no increase in abscess or draining tunnel count from baseline. Patient-reported outcomes (PROs) for comparison included Patient Global Assessment of Worst Skin Pain (PGA-WSK), Patient Global Assessment of Average Skin Pain (PGA-ASK), and the Dermatology Life Quality Index (DLQI). The PGA-WSK and PGA-ASK were single-item numeric scales assessing pain at its worst and pain on average over the past 24 hours, ranging from 0 indicating “no skin pain” to 10 indicating “worst skin pain imaginable.” The DLQI questionnaire is designed for use in adults with skin diseases and is aimed at evaluating how symptoms and treatment affect participants’ health-related quality of life (HRQoL).^16,17,18^ Details of ClinRO and PRO measures can be found in Supplement 1.

### Statistical Analysis

Analyses were performed on the full analysis set population consisting of all randomized participants receiving at least 1 dose of the investigative medicinal product and who have a valid measurement of the primary efficacy variable at baseline and at least 1 postbaseline efficacy assessment. Analyses were conducted at the study population level; thus treatment group allocation was not considered in any of the analyses. All statistical analyses involving an association of data between 1 or more measures included only records where data were not missing for all measures. Analysis was conducted from April 2, 2021 to September 30, 2022, using SAS statistical software (version 9.4, SAS Institute).

### Convergent vs Divergent Validity

Convergent or discriminant validity denotes the degree to which a measure is associated with other measures or variables conceptually or is based on the expected relationship with the chosen variable(s). In particular, convergent validity refers to the relationship between measures that are expected to be highly or more strongly correlated based on similarity of content. In contrast, divergent validity relates to the lack of, or a weaker, association between measures that are not expected to be highly correlated given their dissimilar content.

The hypothesis was that HS-IGA is more strongly associated with other measures of HS severity (HiSCR scores indicating 50%, 75%, and 90% reduction from baseline [HiSCR-50/75/90], IHS4, and HS-PhGA), and less strongly correlated with measures of related but different patient-reported constructs, such as QoL, DLQI, and pain (PGA-WSK NRS and PGA-ASK NRS).

Convergent vs divergent validity was assessed by the correlation between scores on comparison measures (ie, IHS4, HS-PhGA, PGA-WSK, PGA-ASK, DLQI) and HS-IGA scores at baseline and week 12. Spearman rank correlation coefficients were calculated at baseline and week 12. Using commonly accepted conventions, correlation coefficient values ranging from 0.10 to 0.29 were classified as weak correlations, from 0.30 to 0.69 as moderate correlations, and from 0.70 to 1.0 as strong correlations.^19,20^

### Test-Retest Reliability

Test-retest reliability is the extent to which a measure yields consistent scores in the same participants each time it is administered over a short period of time (eg, between 2 days and 2 weeks) and when no change is expected in the concept being measured. The HS-IGA score test-retest reliability was assessed using the correlation between screening and baseline visit data. Intraclass correlation coefficients (ICCs) were calculated. Values of ICC lower than 0.60, 0.60 to 0.69, 0.70 to 0.79, and 0.80 to 1.0 were considered indicative of poor, moderate, good, and very good, respectively, test-retest reliability.^21^

### Responsiveness

Responsiveness or sensitivity to change is the ability of an instrument to measure any degree of change when a known change in the concept of interest has occurred. This assessment of the ability of the HS-IGA score to detect change was evaluated using HiSCR as an anchor to define subgroups of participants reflecting change from baseline to week 12. The HS-IGA was also compared with HiSCR-75 (75% reduction from baseline) and HiSCR-90 (90% reduction from baseline), which are more stringent measures of disease response. Thus, proportions of HS-IGA and HiSCR-50/75/90 responders and nonresponders at week 12 were compared using contingency tables with χ^2^ test of independence.

### Predictive Validity

The ability of HS-IGA to predict response defined according to various anchors was assessed using mixed effects logistic regression models including a random effect for each participant, as well as fixed effects for time, HS-IGA score, interaction between time and HS-IGA score, baseline age and sex, and Hurley stage. Predictiveness is assessed via area under the curve (AUC) of the receiver operating characteristic (ROC) analysis. Strong predictive validity was expected with response criteria based on clinical assessment of HS severity, ie, HiSCR-50/75/90 responder and HS-PhGA clear or minimal responder.

### Patient Centeredness

Patient centeredness of HS-IGA was assessed by the association of HS-IGA with PROs measuring the experience of patients with HS. Thus, PGA-WSK score, PGA-ASK score, DLQI total score, DLQI total score of 1 lower, and DLQI MCID responder rates at week 12 were compared between HS-IGA responders and nonresponders using the Mann-Whitney *U* test. Contingency tables with χ^2^ test results were also provided for the derived DLQI total score of 1 or lower and DLQI MCID response.

## Results

### Cohort Demographics and Baseline Characteristics

A total of 88 patients were randomized and dosed in the UCB HS0001 study. Baseline demographics, disease characteristics, ClinRO, and PRO measures are shown in Table 2.

**Table 2.** Cohort Demographics and Baseline Characteristics

Characteristic	Full analysis set population, No. (%)
No.	88
Age, mean (SD), y	36.7 (12.0)
Sex	
Female	61 (69.3)
Male	27 (30.7)
Race and ethnicity	
American Indian or Alaska native	0
Asian	4 (4.5)
Black or African American	20 (22.7)
Native Hawaiian or other Pacific islander	0
White	61 (69.3)
Other or mixed	3 (3.4)
Country	
Australia	19 (21.6)
Belgium	1 (1.1)
Denmark	3 (3.4)
Germany	8 (9.1)
Norway	2 (2.3)
Russia	11 (12.5)
United States	44 (50.0)
Hurley stages	
2	43 (48.9)
3	45 (51.1)
HS-IGA, mean (SD)	3.8 (1.44)
HS-PhGA	
Minimal	0
Mild	1 (1.1)
Moderate	28 (31.8)
Severe	4 (4.5)
Very severe	55 (62.5)
HS-PhGA, mean (SD)	4.3 (0.96)
IHS4, mean (SD)	43.1 (30.12)
PGA-WSK, mean (SD)	5.2 (2.77)
PGA-ASK, mean (SD)	4.1 (2.53)
DLQI total score, mean (SD)	12.6 (7.49)
DLQI total score ≤1 responders	17 (21.52)
DLQI MCID responders	45 (56.96)
HiSCR-50	42 (53.16)
HiSCR-75	29 (36.71)
HiSCR-90	17 (21.52)

Abbreviations: DLQI, Dermatology Life Quality Index; HiSCR, Hidradenitis Suppurativa Clinical Response (HiSCR scores indicating 50%, 75%, and 90% reduction from baseline [HiSCR-50/75/90]); HS-IGA, Hidradenitis Suppurativa Investigator Global Assessment; HS-PhGA, HS Physician Global Assessment; IHS4, International Hidradenitis Suppurativa Severity Score System; PGA-WSK, Patient Global Assessment of Worst Skin Pain; PGA-ASK, Patient Global Assessment of Average Skin Pain.

### Convergent vs Divergent Validity

The HS-IGA score showed strong convergent validity with IHS4 and HS-PhGA scores at baseline (Spearman correlation, 0.86; 95% CI, 0.79-0.90; *P* < .001, and 0.74; 95% CI, 0.63-0.82; *P* < .001, respectively) and at week 12 (Spearman correlation, 0.73; 95% CI, 0.60-0.82; *P* < .001, and 0.64; 95% CI, 0.49-0.75; *P* < .001, respectively).

Correlation coefficients between HS-IGA and DLQI total scores were 0.15 (95% CI, −0.06 to 0.35; *P* = .16) and 0.20 (95% CI, −0.03 to 0.40; *P* = .08) at baseline and week 12, respectively. Correlation coefficients between HS-IGA and both PGA-WSK and PGA-ASK were less than 0.4 at baseline and at week 12. Supplement 1 describes results from Spearman correlation tests between the HS-IGA and anchor ClinRO and PRO measures at baseline and at week 12.

### Reliability

Complete information for HS-IGA at screening and baseline was available for 87 patients. The ICC between HS-IGA scores at screening and at baseline was 0.92 (95% CI, 0.88-0.95). There was a participant-level variability of 1.91 and residual variability of 0.17.

### Responsiveness

Significant results were obtained when assessing sensitivity of HS-IGA response to the response in each of the HiSCR end points at week 12. Among HS-IGA responders, 85.7% (24/28) achieved HiSCR compared with 35.3% of HS-IGA nonresponders (18/51). Likewise, 67.9% of HS-IGA responders (19/28) achieved HiSCR-75 compared with 19.6% of HS-IGA nonresponders (10/51). With respect to HiSCR-90, only 50% of responders (14/28) were also HS-IGA responders; however, 94.1% of all HS-IGA nonresponders (48/51) were also HiSCR-90 nonresponders (Table 3).

**Table 3.** Responsiveness: Contingency Table of HiSCR and HS-IGA Responses at Week 12

Variable	HS-IGA, No. (%)		χ^2^ test	*P* value
	No	Yes		
**HiSCR-50**				
No	33 (64.7)	4 (14.3)	χ^2^_1,79_ = 18.45	<.001
Yes	18 (35.3)	24 (85.7)		
**HiSCR-75**				
No	41 (80.4)	9 (32.1)	χ^2^_1,79_ = 18.11	<.001
Yes	10 (19.6)	19 (67.9)		
**HiSCR-90**				
No	48 (94.1)	14 (50.0)	χ^2^_1,79_ = 20.83	<.001
Yes	3 (5.9)	14 (50.0)		

Abbreviations: HiSCR, Hidradenitis Suppurativa Clinical Response (HiSCR scores indicating 50%, 75%, and 90% reduction from baseline [HiSCR-50/75/90]); HS-IGA, Hidradenitis Suppurativa Investigator Global Assessment.

### Predictive Validity

A 1-point reduction in HS-IGA score was associated with increased probabilities of being a responder to HiSCR (OR, 0.11; 95% CI, 0.05-0.23), HiSCR-75 (OR, 0.07; 95% CI, 0.03-0.19), HiSCR-90 (OR, 0.13; 95% CI, 0.04-0.35), HS-PhGA clear or minimal (OR, 0.08; 95% CI, 0.03-0.21), and DLQI total score of 1 or lower (OR, 0.39; 95% CI, 0.16-0.90).

The AUCs of HS-IGA score as predictor of HiSCR-50/75/90 at week 12 were 0.69 (95% CI, 0.58-0.81), 0.73 (95% CI, 0.61-0.84), and 0.85 (95% CI, 0.77-0.94), respectively, and using best cut points determined via the Youden index, observed sensitivities/specificities were 0.48/0.84, 0.76/0.60, and 0.76/0.79, respectively. The AUC of HS-IGA score as predictor of HS-PhGA clear or minimal response was 0.71 (95% CI, 0.59-0.84), with a sensitivity of 0.58 and specificity of 0.79 at the best cut point.

### Patient Centeredness

Results for patient centeredness analyses are described in Table 4. Results of χ^2^ testing for independency between a DLQI total score of 1 or less and DLQI MCID responders with HS-IGA at week 12 were nonsignificant. Mann-Whitney *U* testing showed no significant differences on DLQI total score, nor on PGA-ASK and PGA-WSK scores, between HS-IGA responders and nonresponders.

**Table 4.** Patient-Centered Results at Week 12

Variable	HS-IGA, No. (%)		χ^2^ test	*P* value
	No	Yes		
**DLQI total score ≤1 responder**				
No	39 (76.5)	23 (82.1)	χ^2^_1,79_ = 0.34	.56
Yes	12 (23.5)	5 (17.9)		
**DLQI MCID responder**				
No	24 (47.1)	10 (35.7)	χ^2^_1,79_ = 0.95	.33
Yes	27 (52.9)	18 (64.3)		
All DLQI MCID tested patients	Mean (SD)		Mann-Whitney U	
No.	51	28	NA	NA
DLQI total score	7.5 (6.23)	7.2 (7.10)	*U* = 11	.70
PGA-WSK	3.6 (2.84)	3.0 (2.91)	*U* = 10	.38
PGA-ASK	2.9 (2.42)	2.1 (2.46)	*U* = 98	.14

Abbreviations: DLQI, Dermatology Life Quality Index; NA, not applicable; PGA-WSK, Patient Global Assessment of Worst Skin Pain; PGA-ASK, Patient Global Assessment of Average Skin Pain.

## Discussion

Hidradenitis suppurativa is complex in its presentation, owing to significant heterogeneity in lesion types, anatomic areas, surface area involved, and flares of activity. This heterogeneity may result in substantial challenges in reliably and feasibly measuring disease activity in clinical trials. Moreover, complexity of existing instruments also limits their adoption in clinical practice.^22^ Accordingly, there is a compelling need to develop simple-to-use measures in HS management that reflect true activity, are responsive, and that raters can use with accuracy and efficiency. The HS-IGA overcomes limitations related to accurate distinction among lesion types and high counting burden with moderate-to-severe disease, both of which are requisite features of existing trial end points.

These analyses conducted on blinded data from a phase 2 randomized, double-blind, placebo-controlled, active reference arm study to assess the efficacy and safety of bimekizumab in adults with moderate-to-severe HS confirmed the psychometric properties of HS-IGA score. The HS-IGA score demonstrated good convergent validity with IHS4 and HS-PhGA and divergent validity with a general skin disease QoL measure and global pain scales. Importantly, HS-IGA showed very strong test-retest reliability, which suggests that raters may use the measure with accuracy. The HS-IGA also showed responsiveness to change when anchored against HiSCR. For responsiveness, the desire is to maximize true-positive and true-negative results compared with the anchor, while minimizing false-positive and false-negative results. The HS-IGA aligned most closely with HiSCR-90, and this was also corroborated by AUC analysis in which HS-IGA was the best predictor of HiSCR-90. Lack of agreement was primarily driven by HS-IGA false-positive results compared with HiSCR-90, and this is hypothesized to stem from the limitation of HiSCR assessment where any increase in abscess or draining tunnel results in nonresponse, despite a large decrease in overall lesion count. Large decreases in overall lesion count, even with slight increases in abscesses or draining tunnels, would be considered a response for HS-IGA and not HiSCR. The HS-IGA score predicted HiSCR-50/75/90 response and HS-PhGA clear or minimal response at week 12. The HS-IGA was also associated with other clinical measures of HS severity as well as changes in disease severity (ie, HS-PhGA and HiSCR).

The HS-IGA as a measure of disease activity showed low predictive validity with DLQI, a general skin disease QoL measure, and changes in PROs were not statistically significant at week 12 for HS-IGA responders vs nonresponders. This was an expected result because PROs focus on broader concepts of HS beyond just disease activity. Accordingly, some but not a strong correlation is expected. Development of a core set of measures that includes capture of such aspects will be necessary for trials in patients with HS. The HiSTORIC group has developed and is testing the Hidradenitis Suppurativa Quality of Life (HiSQOL) score, which is the first disease-specific PRO to evaluate QoL.^23^

Herein, we have described a validation assessment of the novel HS-specific IGA for use as a disease severity and response measure in interventional trials. The HS-IGA uses the familiar construct of a 6-point dichotomous scale with response defined as 2-point improvement from baseline. The score is based on objective lesion counts, although it limits investigator counting to 21 qualifying lesions. The HS-IGA does not exclude fistulas, nor does it weight particular lesion types. The score includes noninflammatory nodules, which HiSTORIC participants have suggested can evolve into inflammatory nodules, and back again, and thus are important to measure. Inclusion of noninflammatory nodules also renders distinction from inflammatory nodules unnecessary, which importantly may also improve accuracy of rating among patients of color in whom erythema may be difficult to discern. Scars, as readily distinguishable from lesions, are not included in the score. Specification of lesion types and distinction between other difficult to discern lesions (ie, inflammatory nodule vs abscess, or draining abscess vs draining fistula) are not required by the investigator, which may support measurement accuracy. The measure also accounts for anatomic regions of involvement, but it simplifies this concept further by aggregating into upper or lower body regions. This is supported by a study^24^ suggesting that subclassification according to upper and lower regions is relevant. These features of the HS-IGA may allow for improved operational performance and ease of rater use in HS trials.

The HS-IGA may also overcome some limitations of existing disease severity and response instruments. With HiSCR and IHS4, lesion counts are limitless and lesion type distinction is required—both of which may contribute to poor operational performances. In an inter-rater agreement and reliability exercise among dermatologists experienced in patients with HS, observed intervals for limits of agreement were wide relative to the ranges of the scales of all of the measurement instruments tested, including HiSCR and IHS4.^9^ In addition, HiSCR response does not permit an increase in abscess or draining fistula count relative to baseline, even when a greater than 50% reduction in total abscess and inflammatory nodule count has been achieved. As an example, a patient with a 75% decrease in abscess and inflammatory nodule count with an increase in 1 draining fistula would be considered an HiSCR nonresponder. However, an increase in any lesion type would not alone disqualify a response as measured by the HS-IGA provided that the overall number of lesions was improved. Although the threshold for HS-IGA response was higher than for the HiSCR, the instrument may also better distinguish active drug from placebo response rates, the latter of which has been unexpectedly high in recent phase 2/3 trials using HiSCR as the end point.^25,26^ Similarly, HS-IGA may be less susceptible to ceiling effects with the development of more efficacious treatments.

Patients with low lesion counts (<6) at baseline cannot achieve response, as defined by a 2-point change on the scale, with the HS-IGA. In recent phase 2 and phase 3 interventional trials for patients with moderate-to-severe HS, patients with as few as 3 abscesses and/or inflammatory nodules across 2 or more regions have met inclusion criteria. Although there is no standardized definition of moderate-to-severe disease, HiSTORIC participants, including HS experts and patients, were in agreement that 3 to 5 lesions were more representative of patients with milder disease in HS. Ensuring that trial cohorts are reflective of those in the clinical setting with respect to moderate-to-severe disease activity will help ensure that approved drugs have the intended effectiveness in practice.

### Limitations

There are limitations to this study that warrant consideration. The HS-IGA showed low convergence with DLQI. Global assessments have been criticized for oversimplifying the multifaceted nature of dermatological conditions. In HS, instruments that rely on the presence or absence of lesions ignore distinct disease aspects that are also critically important to patients, including symptoms such as pain, drainage, and odor. Moreover, comorbid conditions, the burden of which is high among patients with HS,^4,27^ also likely confound patients’ perception of life quality, even when disease activity is improved. For this reason, ClinROs may not converge with PRO concepts, which are distal to direct measurement of disease manifestations in patients with HS, particularly at only the 12- to 16-week time point. Indeed, HiSCR and IHS4 have also shown modest convergence with DLQI.^13,14,28,29^ Although data from a phase 2 trial were used to assess the psychometric properties, some subgroups had smaller numbers of patients for analysis, which may also have influenced patient-centeredness performance. Nonetheless, the proposed HS-IGA is designed to complement PROs to ensure that all relevant aspects of this multifaceted disease deemed important to patients, experts, and other stakeholders are assessed.^8^

## Conclusions

In this retrospective analysis of a phase 2 randomized double-blind, placebo-controlled, active-reference arm trial, the HS-IGA demonstrated good psychometric properties compared with existing measures and may be considered for use as an end point in clinical trials for HS. The HS-IGA represents a valuable ClinRO to drug development efforts on behalf of patients with HS.

## Notes

Supplement 1. eTable 1. Categorization of anatomic sites to Upper and Lower Body Regions in the HS-IGA score Hidradenitis Suppurativa Clinical Response (HiSCR) International Hidradenitis Suppurativa Severity Score System (ISH4) eTable 2. HS Physician’s Global Assessment (HS-PhGA) Dermatology Life Quality Index (DLQI) Patient's Global Assessment of skin pain: worst (PGA-WSK) and average (PGA-ASK) eTable 3. Spearman correlation between anchors and HS-IGA at baseline and week 12 as measures of convergent or divergent validity eReferences

Supplement 2. Data Sharing Statement
