# Supplementary material for: Validation of the Hidradenitis Suppurativa Investigator Global Assessment: A Novel Hidradenitis Suppurativa–Specific Investigator Global Assessment for Use in Interventional Trials
Source: JAMA Dermatol. 2023 Apr 26;159(6):606–12. doi: 10.1001/jamadermatol.2023.0797 (PMC10134037; doi:10.1001/jamadermatol.2023.0797)
Supplement: Supplement 1. — eTable 1. Categorization of anatomic sites to Upper and Lower Body Regions in the HS-IGA score Hidradenitis Suppurativa Clinical Response (HiSCR) International Hidradenitis Suppurativa Severity Score System (ISH4) eTable 2. HS Physician’s Global Assessment (HS-PhGA) Dermatology Life Quality Index (DLQI) Patient's Global Assessment of skin pain: worst (PGA-WSK) and average (PGA-ASK) eTable 3. Spearman correlation between anchors and HS-IGA at baseline and week 12 as measures of convergent or divergent validity eReferences [file jamadermatol-e230797-s001.pdf]

Supplemental Online Content

Garg A, Zema C, Ciaravino V, et al. Validation of the hidradenitis suppurativa investigator global assessment. *JAMA Dermatol*. Published online April 26, 2023.
doi:10.1001/jamadermatol.2023.0797

eTable 1. Categorization of anatomic sites to Upper and Lower Body Regions in the HS-IGA score

Hidradenitis Suppurativa Clinical Response (HiSCR)

International Hidradenitis Suppurativa Severity Score System (ISH4)

eTable 2. HS Physician's Global Assessment (HS-PhGA)

Dermatology Life Quality Index (DLQI)

Patient's Global Assessment of skin pain: worst (PGA-WSK) and average (PGA-ASK)

eTable 3. Spearman correlation between anchors and HS-IGA at baseline and week 12 as measures of convergent or divergent validity

eReferences

This supplemental material has been provided by the authors to give readers additional information about their work.

© 2023 American Medical Association. All rights reserved.

Supplement

eTable 1. Categorization of anatomic sites to Upper and Lower Body Regions in the HS-IGA score[1]

Anatomic site	Region
Abdomen Fold	Upper
Abdomen Lower	Upper
Abdomen	Upper
Abdominal Fold	Upper
Abdominal	Upper
Axillary (Armpit)	Upper
Back Between Blades	Upper
Back Between Shoulder Blades	Upper
Back, Interscapular	Upper
Back	Upper
Breast	Upper
Cheek	Upper
Chest	Upper
Chin	Upper
Face	Upper
Flank	Upper
Genital	Lower
Gluteal – Buttocks	Lower
Gluteal - Perianal / Perineal	Lower
Gluteal Cleft	Lower

© 2023 American Medical Association. All rights reserved.

Hip Lateral	Lower
Infranasal Middle	Upper
Infranasal	Upper
Inguinal Excl Genital And Pubic Area	Lower
Inguinal Incl Genital And Pubic Area	Lower
Intermammary	Upper
Leg	Lower
Lower Abd	Lower
Lower Abdomen	Lower
Lower Back	Lower
Medial Thigh	Lower
Neck	Upper
Post Auricular Ear	Upper
Posterior Neck	Upper
Posterior/Neck	Upper
Rima Internates	Lower
Sacrum	Lower
Scalp	Upper
Scrotal Sac	Lower
Scrotum	Lower
Submammary	Upper
Supra Pubic	Lower
Thigh Lateral	Lower
Thigh	Lower

© 2023 American Medical Association. All rights reserved.

Upper Back	Upper
Upper Neck/Beard	Upper

Hidradenitis Suppurativa Clinical Response (HiSCR)[2]

The $\mathrm{HiSCR}_{50/75/90}$ is defined as at least a 50/75/90% reduction in the total abscess and inflammatory nodule count from baseline, with no increase in abscess or draining tunnel count from baseline.

International Hidradenitis Suppurativa Severity Score System (ISH4)[3]

The ISH4 assess HS severity by counting the total abscesses, inflammatory nodules, and draining tunnels according to the following equation:

$$IHS4 = (number\ of\ inflammatory\ nodules \times 1) + (number\ of\ abscesses \times 2) + (number\ of\ draining\ tunnels \times 4)$$

A score of 4–10 and ≥11 signifies moderate and severe disease, respectively.

eTable 2. HS Physician's Global Assessment (HS-PhGA)[4]

The HS-PhGA is a 6-point nominal scale based on total abscess, inflammatory and non-inflammatory nodule, and draining fistula count across all affected body regions:

**Category**	**Total Lesion Counts**
Clear	0 abscesses; 0 draining fistulas; 0 inflammatory nodules; 0 non-inflammatory nodules
Minimal	0 abscesses; 0 draining fistulas; 0 inflammatory nodules; ≥1 non-inflammatory nodules
Mild	0 abscesses; 0 draining fistulas; 1-4 inflammatory nodules; or 1 abscess or draining fistula and 0 inflammatory nodules

© 2023 American Medical Association. All rights reserved.

Moderate	0 abscesses; 0 draining fistulas; ≥5 inflammatory nodules; or 1 abscess or draining fistula and ≥1 inflammatory nodule; or 0 abscesses; 2-5 abscesses or draining fistulas and <10 inflammatory nodules
Severe	2-5 abscesses or draining fistulas and ≥10 inflammatory nodules
Very Severe	>5 abscesses or draining fistulas

A response on the HS-PhGA is defined as at least a 2-category change from baseline.

Dermatology Life Quality Index (DLQI)[5]

The DLQI is a validated skin disease-specific questionnaire aimed at the evaluation of how symptoms and treatment affect patients' health related QOL. It includes 10 items about symptoms and feelings, daily activities, leisure, work and school, personal relationships, and treatment. Each item is scored from 0 to 3, with 3 representing the worst state. A DLQI total score is obtained by adding the 10 item scores, which ranges from 0 to 30. Higher score indicate lower health related QOL. A 4-point improvement in DLQI total score (DLQI response) has been reported to be meaningful for the participant (within participant minimal important difference), while DLQI total absolute score of 0 or 1 indicates no or small impact of the disease on HRQoL.

Patient's Global Assessment of skin pain: worst (PGA-WSK) and average (PGA-ASK)

The Patient's Global Assessment of worst skin pain (PGA-WSK) and Patient's Global Assessment of average skin pain (PGA-ASK) utilize a single item 11-point numeric rating scale with 0 indicating "no skin pain" to 10 indicating "worst skin pain imaginable". Worst skin pain is assessed by the patient as "pain at its worst during the last 24 hours", and average skin pain is assessed as "pain on average in the last 24 hours."

© 2023 American Medical Association. All rights reserved.

eTable 3. Spearman correlation between anchors and HS-IGA at baseline and week 12 as a measure of convergent or divergent validity.

	**Baseline**		**Week 12**	
**Anchor**	**Correlation**	**p-value**	**Correlation**	**p-value**
IHS4	0.86	<0.001	0.73	<0.001
HS-PhGA	0.74	<0.001	0.64	<0.001
DLQI Total Score	0.15	0.156	0.20	0.082
PGA-WSK	0.12	0.284	0.28	0.014
PGA-ASK	0.11	0.322	0.29	0.009

© 2023 American Medical Association. All rights reserved.

eReferences:

1. Garg A, Zema C, Kim K, Gao W, Chen N, Jemec GBE, Kirby J, Thorlacius L, Villumsen B, Ingram JR. Development and initial validation of the HS-IGA: a novel hidradenitis suppurativa-specific investigator global assessment for use in interventional trials. *Br J Dermatol.* 2022;187(2):203-210.
2. Kimball AB, Jemec GEB, Yang M et al. Assessing the validity, responsiveness and meaningfulness of the hidradenitis suppurativa clinical response (HiSCR) as the clinical endpoint for hidradenitis suppurativa treatment. *Br J Dermatol.* 2014;171:1434–1442.
3. Zouboulis CC, Tzellos T, Kyrgidis A, et al. European Hidradenitis Suppurativa Foundation Investigator Group. Development and validation of the International Hidradenitis Suppurativa Severity Score System (IHS4), a novel dynamic scoring system to assess HS severity. *Br J Dermatol*. 2017;177(5):1401-1409.
4. Kimball AB, Kerdel F, Adams D, et al. Adalimumab for the treatment of moderate to severe Hidradenitis suppurativa: a parallel randomized trial. *Ann Intern Med*. 2012;157(12):846-855.
5. Finlay AY, Khan GK. Dermatology Life Quality Index (DLQI)--a simple practical measure for routine clinical use. *Clin Exp Dermatol*. 1994;19(3):210-216.

© 2023 American Medical Association. All rights reserved.
