# Supplementary material for: Validation of the Hidradenitis Suppurativa Investigator Global Assessment: A Novel Hidradenitis Suppurativa–Specific Investigator Global Assessment for Use in Interventional Trials
Source: JAMA Dermatol. 2023 Apr 26;159(6):606–12. doi: 10.1001/jamadermatol.2023.0797 (PMC10134037; doi:10.1001/jamadermatol.2023.0797)
Supplement: Supplement 2. — Data Sharing Statement [file jamadermatol-e230797-s002.pdf]

Data Sharing Statement

Garg. Validation of the Hidradenitis Suppurativa Investigator Global Assessment. *JAMA Dermatol*. Published April 26, 2023. doi:10.1001/jamadermatol.2023.0797

Data
Data available: Yes
Data types: Other (please specify)
Additional Information: Statistical analysis plan, data dictionary
How to access data: amgarg@northwell.edu
When available: With publication

Supporting Documents
Document types: None

Additional Information
Who can access the data: NA
Types of analyses: for specified purpose
Mechanisms of data availability: after approval of proposal
Any additional restrictions: NA
